# Retraction Announcement

**Published:** 2016

**Pathological changes in the maxillary sinus mucosae of patients with recurrent odontogenic maxillary sinusitis.**

This article titled “Pathological changes in the maxillary sinus mucosae of patients with recurrent odontogenic maxillary sinusitis^1^” published in Pak J Med Sci. 2014;30(5):972-975. is being retracted. After publication it was found to be plagiarized from an article titled “Structural and functional changes in the mucosa of the maxillary sinus with recurrent odontogenic sinusitis” published in a Russian Journal “The Institute of Dentistry”. 2011 Number 4.^2^ Thus on grounds of plagiarism the concerned article is being retracted.

Shaukat Ali Jawaid

Chief Editor

Pakistan Journal of Medical Sciences

Karachi - Pakistan.

## References

[1] Feng L, Li H, Ling-Ling E, Li CJ, Ding Y (2014). Pathological changes in the maxillary sinus mucosae of patients with recurrent odontogenic maxillary sinusitis. Pak J Med Sci.

[2] “Structural and functional changes in the mucosa of the maxillary sinus with recurrent odontogenic sinusitis” was in the Russian magazine “The Institute of Dentistry“. 2011 number 4

